# Phenolic Compound Analysis and Pharmacological Screening of *Vitex agnus-castus* Functional Parts

**DOI:** 10.1155/2021/6695311

**Published:** 2021-07-21

**Authors:** Assia Berrani, Ilias Marmouzi, Abdelhakim Bouyahya, Mourad Kharbach, Maha El Hamdani, Meryem El Jemli, Aicha Lrhorfi, Meryem Zouarhi, My El Abbes Faouzi, Rachid Bengueddour

**Affiliations:** ^1^Laboratory of Biochemistry, Biotechnology, Health and the Environment, Department of Biology, Faculty of Sciences, Ibn Tofail University, Kenitra, Morocco; ^2^Université Mohammed V de Rabat, Faculté de Médecine et de Pharmacie, Laboratoire de Pharmacologie et Toxicologie, Rabat, Morocco; ^3^Laboratory of Human Pathologies Biology, Faculty of Sciences and Genomic Center of Human Pathologies, Faculty of Medicine and Pharmacy, Mohammed V University in Rabat, Morocco; ^4^Faculté des Sciences, Université Ibn Tofail, Rabat, Morocco; ^5^Faculty of Pharmacy, Mohammed VI University of Health Sciences (UM6SS), Casablanca, Morocco; ^6^Laboratory of Electrochemistry and Materials Environment (LMEE), Department of Chemistry, Faculty of Sciences, Ibn Tofail University, Kenitra, Morocco

## Abstract

*Vitex agnus-castus* is a medicinal plant of the *Verbenaceae* family, widely used in traditional medicine. This study is aimed at investigating the functional variability of phenolic compounds in different parts (leaves, stems, flowers, roots, and seeds) of *Vitex agnus-castus* methanolic extracts and at assessing their *in vitro* antidiabetic, antioxidant, and antibacterial activities. The results of HPLC-DAD-QTOF-MS indicated the presence of 25 phenolic compounds with a remarkable variability between plant parts; high levels were registered in chlorogenic, vanillic, 3,4-dihydroxybenzoic, and 3-hydroxybenzoic acids; hesperidin; and luteolin. *V. agnus castus* fruits and stems presented higher antioxidant activities. The extracts inhibited the growth of five pathogenic bacteria with MIC values documented between 7.81 and 31.25 mg/mL. *In vitro* antihyperglycemic effect revealed higher effect in flowers (2921.84 *μ*g/mL) and seeds (2992.75 *μ*g/mL) against *α*-glucosidase and of leaves (2156.80 *μ*g/mL) and roots (2357.30 *μ*g/mL) against *α*-amylase. The findings of this showed that *V. agnus castus* is a promising source for antidiabetic bioactive compounds. However, further investigations regarding the evaluation of *in vivo* antidiabetic effects of these compounds are needed.

## 1. Introduction

*Vitex agnus-castus* L, the chaste tree, is a shrub belonging to the *Vitex* genus of the *Verbenaceae* family. It is scattered on the shores and on the shores of the Mediterranean and Asia. *V. agnus castus* is a deciduous plant that reaches heights up to 5 m [1]. The leaves are opposite, in the form of a hand, composed of five to seven radiating leaves which are carried on a main stem. The leaves are linear, lanceolate, toothed, dark green above, and gray under a very tight felt. From August to October, panicles of flowers with many flowers form. The flowers are fragrant and of a blue, lilac, pink, or white color. The berries resemble peppercorns, hard, violet to black, yellowish in the interior, half covered with their green calyces, and containing four seeds. The smell is aromatic and spicy, the taste warm and special. The parts used today as herbal medicine are the ripe dried berries.

*V. agnus castus* is used in traditional medicine as digestive aid, for stomachache, antidiabetic, and antispasmodic [2]. Moreover, this species is widely used for the treatment of premenstrual syndrome and premenstrual dysphoric disorder [3]. *V. agnus castus* contains a variety of chemical families such as flavonoids, terpenoids, and iridoids; those compounds are present essentially in fruits, while leaves are rich in polyphenols. Essential oils (EO) of *V. agnus castus* contain diterpene constituents such as vitexilactone, rotundifuran, and vitexlactam A [4, 5]. Moreover, oxygenated compounds such as bornyl acetate, 1,8-cineol, limonene, *α*-pinene, and *β*-pinene are also found in *V. agnus castus* (EO). Furthermore, *O*-diphenol is the chemotype of polyphenolic compounds, whereas casticin, chrysosplenetin, chrysosplenol D, cynaroside, isorhamnetin, luteolin, and isoorientin are the main flavonoid components of *V. agnus castus* extracts [2, 6, 7].

Recently, essential oils and organic extracts from *V. agnus castus* showed promising properties including antibacterial, antioxidant, anticancer, and anticorrosion [7–11]. However, at our knowledge, there is no study that investigated a comparison between different plant parts of *V. agnus castus*. Therefore, the aims of this study are to determine the phenolic compounds of *V. agnus castus* methanolic extract from the leaves, stems, flowers, roots, and seeds using HPLC-DAD-QTOF-MS analysis and to investigate their functional properties (antioxidant, antidiabetic, and antibacterial).

## 2. Experimental Procedures

### 2.1. Collection and Identification of the Plant

*Vitex agnus-castus* was harvested at the edge of Oued Baht Caidat of Al Mokhtar, Sidi Al Kamel of the province of Sidi Kacem which corresponds to the following coordinates: latitude 34°13′00^″^North, longitude 5°42′00^″^West, altitude 194 m. The plant was collected in August 2014 at the time of their flowering stage. The samples (leaves, roots, stems, flowers, and seeds) were rinsed, dried in a dry place, aired in the absence of light, and finally crushed using a grinder. The species was identified the Prof. Zidane Lahcen (Département Biologie, Productions Végétales Animales et Agro-Industrie, Ibn Tofail University).

### 2.2. Extraction and Phenolic Compound Quantification

The extraction of the various parts was carried out under cold conditions by maceration of 5 g of the vegetable powder in 40 mL of methanol in solution for 24 hours with stirring. The filtrates obtained were placed in closed bottles and then stored in the fridge (put temp 4-7°C). The extract concentration was calculated relatively to the plant material and used for all assays. The different parts of the plant were assessed for polyphenolic contents. Total polyphenol content was determined by the Folin-Ciocalteu method [12], the flavonoids by the aluminum chloride (AlCl_3_) colorimetric method, and the tannins condensed by the sulfuric vanillin reaction. The total polyphenol content was expressed in mg gallic acid equivalent/g extract (mg GE/g extract), the total flavonoid content in quercetin equivalent mg/g extract (mg QE/g extract), and condensed tannins in mg catechin equivalent/g extract (mg CE/g extract).

### 2.3. Chromatographic Analysis

Phenolic compound analysis was performed using HPLC-DAD-QTOF-MS [13]. The system consisted of a binary pump (G1312A, Agilent Technologies, Inc., Wilmington, DE, USA) and an auto sampler (G1330B, Agilent Technologies, Inc. Wilmington, DE, USA) coupled to a mass spectrometer equipped with an electrospray ionizer source (MS; ESI-; Micro mass Quattro Micro; Waters, Milford, MA, USA). Reversed phase HPLC separation was carried out using a zorbax C18 column Zorbax (100 mm × 2.1 mm × 1.7 *μ*m, Agilent Technologies, Santa Clara, CA, USA). The mass spectrometer was operated in negative ion mode with the following parameters: capillary voltage, 3.0 kV; cone voltage, 20 V; and extractor, 2 V. Source temperature was 100°C, desolvation temperature was 350°C, cone gas flow was 30 L/h, and desolvation gas flow was 350 L/h. The mobile phase components were 0.1% formic acid (A) and acetonitrile with 0.1% formic acid (B). The mobile phase gradient was as follows: 0 min, 90% A; 0–18 min, 30% A; 18–20 min, 30% A; 20–23 min, 30% A; 23–25 min, 90% A; and 25–30 min, 90% A. The injection volume was 10 *μ*L, and the column temperature was 35°C. The flow rate of the mobile phase was 0.5 mL/min. The phenolic acids were identified on the basis of their retention times, MS spectra, and molecular-ion identification.

### 2.4. *α*-Amylase Inhibition

In *α*-amylase inhibition assay, 250 *μ*L of the sample was mixed with 250 *μ*L of *α*-amylase (240 U/mL, in 0.02 M phosphate buffer, pH 6.9, with 0.006 M NaCl). After incubating at 37°C for 10 min, 250 *μ*L of 1% (*w*/*v*) soluble starch (in 0.02 M phosphate buffer, pH 6.9) was added and the mixture was further incubated at 37°C for 30 min, followed by adding 500 *μ*L of DNS color reagent, and stopped by heating in boiling water bath for 10 min. After cooling to room temperature, the mixture was diluted with 2 mL of buffer and the absorbance was measured at 540 nm. The inhibition percentage (%) was calculated by the following equation for both enzymatic assays:

(1) $$\begin{array}{c} \text{Inhibition }\left(\%\right)=\frac{\left(\text{AC}-\text{ACb}\right)-\left(\text{AS}-\text{ASb}\right)}{\text{AC}-\text{ACb}}\times 100, \end{array}$$

where AC is the absorbance of the control, ACb is the control blank, AS is the absorbance of the sample, and AS is the absorbance of the sample blank.

### 2.5. *α*-Glucosidase Inhibition

The *α*-glucosidase enzyme (0.1 U/mL) and substrate *p*-nitrophenyl-*α*-D-glucopyranoside (*p*-NPG, 1 mM) were dissolved in potassium phosphate buffer (0.1 M, pH 6.7), and all samples were dissolved in distilled water. The inhibitor (150 *μ*L) was preincubated with the enzyme (100 *μ*L) at 37°C for 10 min, and then, the substrate (200 *μ*L) was added to the reaction mixture. The enzymatic reaction was performed at 37°C for 30 min. The reaction was then terminated by the addition of Na_2_CO_3_ (1 M, 1 mL). All samples were analyzed in triplicate with different concentrations to determine the IC_50_ values, and the absorbance was recorded at 405 nm [13].

### 2.6. Free Radical Scavenging Activity

The free radical scavenging activity of the extracts was measured by 1.1-diphenyl-2-picrylhydrazil (DPPH) as described by Huang et al. [14]. Briefly, 0.2 mm solution of DPPH in methanol was prepared and 0.5 mL of this solution was added to 2.5 mL of plant extract and was allowed to stand at room temperature for 30 min, and then, absorbance was read at 517 nm against blank samples. Lower absorbance of the reaction mixture indicated higher free radical scavenging activity. The radical-scavenging activity (RSA) was calculated as a percentage of DPPH discoloration. The following equation was used:

(2) $$\begin{array}{c} \%\text{RSA}=\left[\frac{\text{AD}\_\text{AE}}{\text{AD}}\right]*100, \end{array}$$

where AD is the absorbance value of the DPPH blank sample and AE is the absorbance value of the test solution. AE was evaluated as the difference between the absorbance value of the test solution and the absorbance value of its blank.

Thin layer chromatography was used to identify the active compounds against DPPH radicals [15]. The chemical revelator is a methanolic solution of DPPH with a concentration of 2 mg/mL for the detection of extracts as well as compounds having antioxidant activity [16]. The frontal ratios of the spots resulting from the separation were calculated.

### 2.7. Ferric Reducing/Antioxidant Power

The ferric ion (Fe^3+^) reducing antioxidant power (FRAP) method was used to measure the reducing capacity of extracts with a slight modification, which involves the presence of extracts to reduce the ferricyanide complex to the ferrous form. The absorbance was measured at 700 nm at the reaction time of 30 min. The reducing power of the extracts was represented as ascorbic acid equivalent (mg AAE/g edw).

### 2.8. Trolox Equivalent Antioxidant Capacity

The Trolox equivalent antioxidant capacity (TEAC) was used. The assay is based on the inhibition of the absorbance of the radical cation 2,2′-azino-bis(3-ethylbenzothiazoline-6-sulfonate) (ABTS+) solution when it is exposed to an antioxidant as described. The ABTS+ cation radical was produced by the reaction between 10 mL of 2 mm ABTS in H_2_O and 100 mL of 70 mm potassium persulfate, stored in the dark at room temperature for 24 h. The ABTS+ solution was then diluted with methanol to obtain an absorbance of 0.70 at *K* = 734 nm and adjusted the temperature to 30°C. Samples were prepared in triplicate by diluting 50 mL of extracts in 2 mL of the ABTS^+^ solution diluted with methanol and left to react for 1 min. Absorbances were recorded at 734 nm. The antioxidant activity samples were expressed as TEAC values, defined as the concentration of standard Trolox with the same antioxidant capacity of the extract under investigation.

### 2.9. Antibacterial Activity

The antimicrobial activity was examined against five bacterial strains: *Staphylococcus aureus* CIP 483, *Bacillus subtilis* CIP 5262, *Escherichia coli* CIP 53126, *Pseudomonas aeruginosa* CIP 82118, and *Salmonella enterica* CIP 8039. For the minimum inhibitory concentration (MIC) and the minimum bactericidal concentration (MBC) of our crude extract, a modified resazurin microtitre-plate assay was used as reported by Sarker et al. [17]. Any color changes from purple to pink or colorless were recorded as positive. The lowest concentration at which color change occurred recorded as the MIC value; the minimum bactericidal concentration (MBC) is the lowest concentration of substance that leaves at most 0.01% of surviving germs [18].

### 2.10. Statistical Analysis

The significance of differences between multiple averages was determined by one-way analysis of variance (ANOVA), followed by Tukey's post hoc test at a 5% significance level.

## 3. Results and Discussion

### 3.1. Chemical Composition

**Figure 1:**
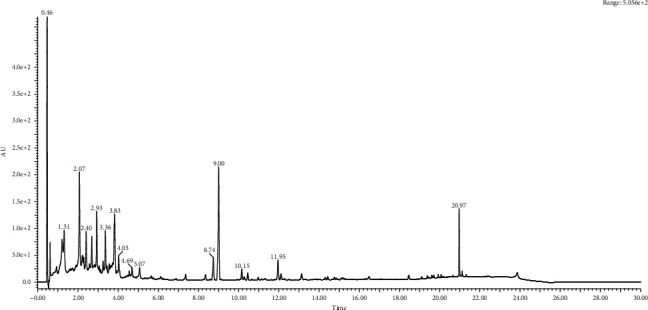
Chromatography analysis of Vitex seed extracts.

**Figure 2:**
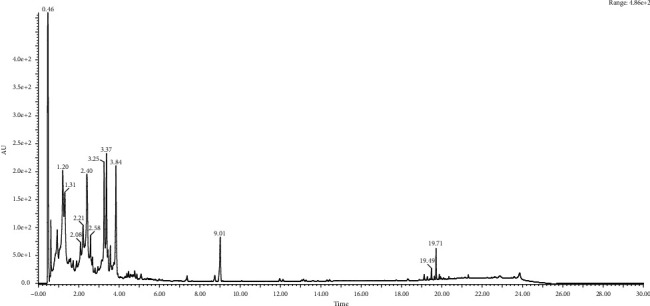
Chromatography analysis of Vitex stem extracts.

**Figure 3:**
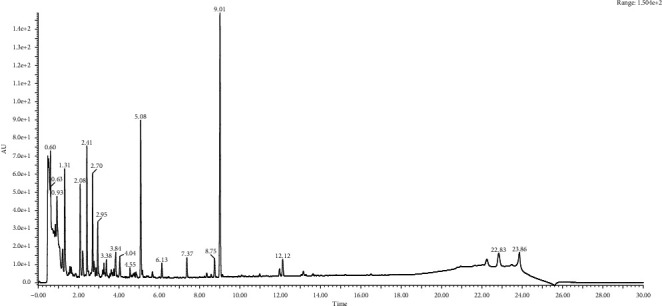
Chromatography analysis of Vitex leaf extracts.

**Figure 4:**
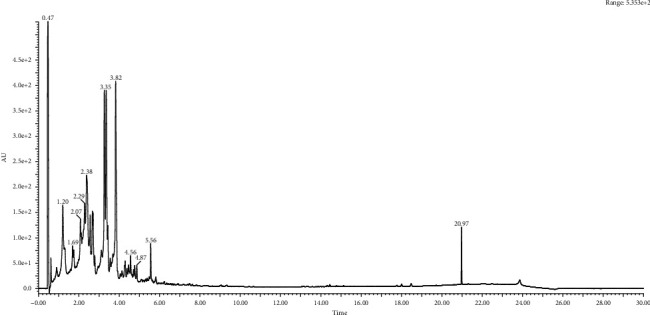
Chromatography analysis of Vitex root extract.

**Figure 5:**
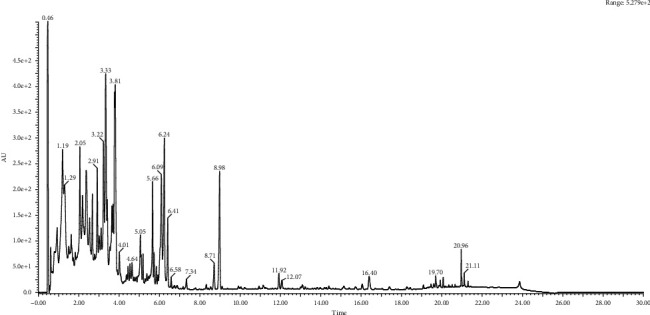
Chromatography analysis of Vitex flower extracts.

**Figure 6:**
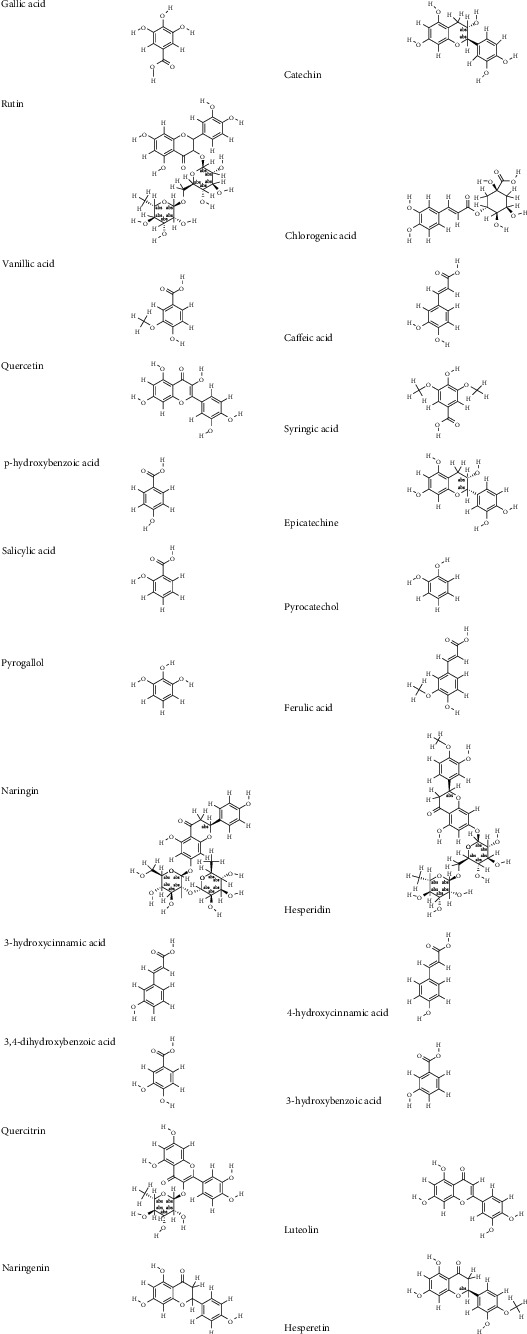
Chemical structures of identified bioactive compounds.

The total phenolic contents of the various parts of the plant were determined and the results obtained are summarized (Figures 1 – 6 and Table 1). The lowest content of polyphenols was that of the stems (4.21 ± 1.53 mg GE/g), while the highest value was that of the flower part (13.11 ± 1.28 mg GE/g). The flavonoid levels followed an inverse trend. The tannin levels ranged between 2.72 ± 0.73 mg CE/g for stems and 9.29 ± 1.47 mg CE/g for flowers. The results of HPLC-DAD-QTOF-MS are shown in Table 2. As listed, *Vitex* extracts showed an important diversity and variability between plant parts. The chlorogenic acid (main phenolic compound) is present in all plant parts in the following concentrations: 42019.9 ± 2269.07 *μ*g/kg (leaves), 295098 ± 15640.2 *μ*g/kg (roots), 213138 ± 11935.7 *μ*g/kg (stems), 259626 ± 13500.6 *μ*g/kg (flowers), and 122900 ± 4547.28 *μ*g/kg (seeds). Moreover, the chemical analysis by HPLC-MS was identified 11 types of flavonoids; the most abundant is luteolin with high levels especially in the flower part with a value of 77047.5 ± 4006.47 *μ*g/kg. Importantly, other previous studies have also revealed numerous phenolic compounds in *V. agnus castus* extracts [6, 19–21]. Indeed, the rate of phenolic compounds found in our results is not exactly similar to those identified in these reposted works. This difference is certainly due the geographical origins of the plant. However, in our study, the variability between chemical compounds in different parts can be explained by the ability of each organ to synthesis secondary metabolites but also to metabolic regulation.

### 3.2. *In Vitro* Antidiabetic Effects

Medicinal plants with antidiabetic properties may be a useful source for effective antidiabetic drugs. In the present research, different extracts of *V. agnus castus* were evaluated for their antidiabetic activity. Two different *in vitro* assays were used to evaluate this activity, *α*-glucosidase and *β*-galactosidase uptake assay. The results of the *in vitro* inhibitory activity against *α*-amylase and *α*-glucosidase enzymes by methanolic extracts of different parts of the plant are stated as the percent inhibition. The inhibition of the two studied enzymes depends on the used concentration. Indeed, at the high concentration, the percentage inhibition of *α*-glucosidase was obtained significantly with leaf part (89.43%), followed by seeds (83.62%), roots (80.09%), flowers (79.38%), and stems (70%), respectively. Moreover, the percentage inhibition of *α*-amylase showed that the root part possessed a high inhibitory activity (94.75%), followed by leaves (83.2%), flowers (75.85%), and seeds (73.83%), respectively. To compare the obtained results, the IC_50_ values were calculated by plotting percentage (%) of inhibition versus extract concentrations (Table 3). As listed, the flowers highly inhibited the *α*-glucosidase with IC_50_ = 2921.84 *μ*g/mL, whereas the *α*-amylase was strongly inhibited by the leaf part (IC_50_ = 2156.80 *μ*g/mL). Moreover, previous studies have indicated the antidiabetic effects of *V. agnus castus* [22]. The antidiabetic action of this plant is not mediated by the inhibition of enzymes involved in hydrocarbon catabolism, while it is related to its capacity to hypoglycemic state *via* hormonal interaction mechanisms [22]. Berrani et al. [9] showed that methanolic extracts of *V. agnus castus* aerial part exhibit a remarkable *in vivo* antidiabetic effect in diabetic rats induced by streptozotocin. Indeed, the administration of this extract at 300 mg/kg induced a decrease in blood glucose after 2 h and improved lipid profile after 21 days [9]. In addition, other plants belonging to other families have exerted important *in vitro* antidiabetic effects *via* the inhibition of *α*-amylase and *α*-glucosidase [23, 24]. These plants contain phenolic compounds similar to those identified in *V. agnus castus* which they are certainly responsible for these effects.

Main bioactive compounds (chlorogenic acid, luteolin, vanillic, hesperidin, and luteolin are the) identified in *agnus castus* are involved certainly in these antidiabetic effects. In fact, literature reports showed that these compounds possess promising *in vitro* and *in vivo* antidiabetic properties with several mechanisms of action. Indeed, several studies reported the antidiabetic effects of luteolin [25–27]. In an *in vitro* study, Kyungpook et al. [27] showed that luteolin exhibits an important inhibition of *α*-glucosidase (36% at 0.5 mg/mL) compared with acarbose used as a standard drug, the inhibition of *α*-amylase was less potent than that of acarbose. Yan et al. [26] reported that luteolin reduces the activity of *α*-glucosidase at a dose dependent-manner (IC_50_ = 1.72 ± 0.05.10^−4^ mol/L). Moreover, this inhibition was mediated by a noncompetitive inhibition action with a single inhibition site on *α*-glucosidase (*K*_*i*_ = 1.40 ± 0.02 × 10^−4^ mol/L) [26]. On the other hand, this compound inhibited the activity of several intestinal enzymes such as *α*-glucosidase, maltase activity, and sucrase in an animal model [25]. Chlorogenic acid showed also remarkable antidiabetic effects [28–30]. *In vitro* studies reported that chlorogenic acid inhibits *α*-amylase and *α*-glucosidase (key enzymes linked to type 2 diabetes) activities in a dose-dependent manner [30]. Nicasio et al. [28] reported that chlorogenic acid decreases plasma glucose surge during the glucose tolerance test in streptozotocin-induced diabetic rats [29]. Karthikesan et al. [31] reported that chlorogenic acid increases the levels of plasma insulin and glycogen, decreases glycosylated hemoglobin, and reverses the altered activities of some enzymes implicated in glucose metabolism such as glucose-6-phosphatase, fructose-1,6-bisphosphatase, glucokinase, and hexokinase in an animal model [31]. Moreover, mechanistic studies revealed that chlorogenic acid exhibits a stimulation of glucose transport in L6 skeletal muscle cells and increases GLUT 4 translocation through AMPK activation [32]. In addition, this acid inhibited the gluconeogenesis through the downregulation of gluconeogenic enzyme G6Pase, stimulates glucose uptake in skeletal muscles by increasing GLUT-4 expression and translocation to plasma membrane, and increases AMPK phosphorylation in time- and dose-dependent manners [32].

### 3.3. Antioxidant Activity

Recently, several scientific investigations revealed that oxidative stress plays an important role in the development of numerous pathologies such as cancer, diabetes, and autoimmune diseases. The antioxidants are the molecules which eliminate these unstable free radicals and therefore inhibit the rate of oxidation and protect cells from damage [33]. These bioactive molecules are mostly used for the prevention and treatment of oxidative stress-related diseases such as diabetes, Alzheimer's disease, and cancer [34]. Several tests have been developed for the analysis of *in vitro* and *in vivo* antioxidant activity [35]. Furthermore, to study the antioxidant activity of our plant extracts, the ability to scavenge the stable free radical DPPH and the cation ABTS and their ferric reducing antioxidant power (FRAP) was evaluated.

According to DPPH assay results (Table 1), the flower part exhibited a high IC_50_value = 0.199 mg/mL, followed by roots (IC_50_ = 0.283 mg/mL), and then stems and leaves. The lowest activity was attributed to seeds with 0.612 mg/mL. The antioxidant effect based on thin layer chromatography revealed inhibitory compounds in the plant parts. These constituents are capable of stabilizing the DPPH radicals. The results of the antioxidant activity evaluated by FRAP assay are shown in Table 1, used as ascorbic acid equivalent reference. We observed that the flowers, roots, and stems (1.347 ± 0.08, 1.293 ± 0.06, and 0.926 ± 0.17 mg AAE/g, respectively) exhibited a high reducing power in comparison to leaves (0.488 ± 0.14 mg AAE/g) and seeds (0.615 ± 0.09 mg AAE/g). The plant part ability to inhibit the ABTS+ radical was obtained using the ABTS assay with Trolox in a calibration reference curve. The discoloration test results are reported in Table 1. As shown, we noted that the roots (27.668 ± 0.46 mg TE/g) had the most active effect compared to other parts. Several other studies have reported the antioxidant effects [36, 37]. These works revealed different findings according to the used methods and type of tested extract.

### 3.4. Antibacterial Activity

The antibacterial activity was evaluated by the microdilution method. The results were expressed by MIC and MBC values. Methanol extracts of different parts showed remarkable antibacterial powers with some variability depending on the part of plant and the tested strain. The MIC and MBC values are listed in Table 4. As summarized, the MIC and CMB obtained ranged between 7.81 and 31.25 mg/mL. Indeed, methanolic extracts of flowers showed the lowest MIC and MBC values against tested strains especially *P. aeruginosa* (MIC = MBC = 7.81). The ration CMB/MIC is reported in Table 4. As shown, methanolic extracts of different parts revealed a bactericidal power by a ratio of CMB/MIC between 1 and 2 (less than 4). Moreover, because of the development of bacterial resistance, infectious diseases became a serious problem of human health. Different sources have been screened for identification and isolation of new bioactive molecules with antibacterial properties. This work revealed that extracts from different parts of *Vitex agnus-castus* inhibited bacterial growth with the lowest concentrations. Other studies have reported the antibacterial effects of *Vitex agnus-castus* [38–40]. Our results are in consonance with those obtained in these reported works with certain difference, which could be related to the difference in chemical compounds, bacterial strains, and used methods. Moreover, the antibacterial action of *Vitex agnus-castus* extracts is most probably due to the major phenolic compounds such as luteolin and chlorogenic acid that show remarkable antibacterial effects [41, 42].

## 4. Conclusion

The present work has highlighted the richness of *Vitex agnus-castus* in phenolic compounds, especially flavonoids and tannins, which are most likely responsible for these biological activities. The results of the present study demonstrated that the methanolic extracts of the different parts of the Vitex plant are effective inhibitors of *α*-amylase and *α*-glucosidase, which can help be of value in lowering postprandial glucose levels. However, the major compounds responsible for the inhibitory action of *α*-amylase and *α*-glucosidase have yet to be characterized, isolated, and tested for *in vitro* and *in vivo* antidiabetic activity. This can be beneficial in the development of new antidiabetic agents from indigenous plant resources.

## Figures and Tables

**Table 1:** Phytochemical analysis and antioxidant activities.

Plant parts	TPC^a^	TFC^b^	CTC^c^	FRAP^d^	DPPH^e^	ABTS^f^	TLC
Leaf	5.50 ± 0.35	1.39 ± 0.17	3.20 ± 2.27	0.488 ± 0.14	0.420 ± 0.01	35.404 ± 29.10	0.86
Root	9.59 ± 0.36	0.67 ± 0.10	7.21 ± 0.43	1.296 ± 0.17	0.283 ± 0.02	27.668 ± 0.46	0.92, 0.86, 0.76, 0.50
Stem	4.21 ± 1.53	0.41 ± 0.01	2.72 ± 0.73	0.923 ± 0.06	0.410 ± 0.01	101.272 ± 17.87	0.94, 0.86, 0.50
Flower	13.11 ± 1.28	1.84 ± 0.01	9.29 ± 1.47	1.347 ± 0.08	0.199 ± 0.001	45.262 ± 22.72	0.84, 0.78, 0.68, 0.52
Seed	5.26 ± 0.44	1, 13 ± 0.20	3.84 ± 1.44	0.615 ± 0.09	0.612 ± 0.007	23.124 ± 4.96	0.86

^a^mg GE/g; ^b^mg QE/; ^c^CE/g; ^d^AAE/g; ^e^ IC_50_; ^f^TE/g. Data are reported as the mean ± standard error (*n* = 3).

**Table 2:** Phenolic composition of different parts of the plant *Vitex agnus-castus* by HPLC-DAD-QTOF-MS in *μ*g/kg.

Compounds	Leaves	Roots	Stems	Flowers	Seeds
Gallic acid	12077.1 ± 652.16	20.20 ± 1.07	908.13 ± 50.86	1587.85 ± 82.57	199.46 ± 7.38
Catechin	0.68 ± 0.04	0.86 ± 0.05	0.89 ± 0.05	0.89 ± 0.05	1.44 ± 0.05
Rutin	0.26 ± 0.02	0.01 ± 0.00	0.18 ± 0.009	0.26 ± 0.013	0.12 ± 0.005
Chlorogenic acid	42019.9 ± 2269.07	295098 ± 15640.2	213138 ± 11935.7	259626 ± 13500.6	122900 ± 4547.28
Vanillic acid	25387.7 ± 1370.93	25008.5 ± 1325.45	31908.7 ± 1786.88	9003.7 ± 468.19	19176 ± 709.51
Caffeic acid	27230.3 ± 1470.44	90979.9 ± 4821.94	111044 ± 6218.47	265546 ± 13808.4	44277.2 ± 1638.25
Quercetin	205.41 ± 11.09	2.38 ± 0.13	119.43 ± 6.69	450.97 ± 23.45	300.27 ± 11.11
Syringic acid	78.17 ± 4.22	12.40 ± 0.66	64.27 ± 3.60	9.28 ± 0.48	18.18 ± 0.67
p-Hydroxybenzoic acid	14878.2 ± 803.42	10937.2 ± 579.67	16377.4 ± 917.13	21984 ± 1143.17	22771 ± 842.53
Epicatechin	nd	nd	nd	nd	83.16 ± 3.08
Salicylic acid	587.77 ± 31.74	458.81 ± 24.32	296.92 ± 16.63	12.65 ± 0.66	448.05 ± 16.58
Pyrocatechol	125.29 ± 6.77	35.32 ± 1.87	744.76 ± 41.71	942.84 ± 49.03	546.54 ± 20.22
Pyrogallol	362.50 ± 19.57	nd	nd	76.26 ± 3.97	nd
Ferulic acid	392.64 ± 21.20	2.95 ± 0.16	525.96 ± 29.45	255.83 ± 13.30	101.52 ± 3.76
Naringin	nd	125.62 ± 6.66	nd	nd	nd
Hesperidin	24325.4 ± 1313.57	nd	nd	nd	20853.4 ± 771.58
3-Hydroxycinnamic acid	1234.27 ± 66.65	3.15 ± 0.17	1249.02 ± 69.94	1005.82 ± 52.30	592.04 ± 21.91
4-Hydroxycinnamic acid	7683.83 ± 414.93	113.72 ± 6.03	12098.4 ± 677.51	8437.54 ± 438.75	7769.79 ± 287.48
3,4-Dihydroxybenzoic acid	29106.6 ± 1571.76	6803.23 ± 360.57	66630.2 ± 3731.29	85725.5 ± 4457.73	53387.9 ± 1975.35
3-Hydroxybenzoic acid	4266.39 ± 230.38	3412.68 ± 180.87	28681.4 ± 1606.16	51478.8 ± 2676.90	19735.2 ± 730.20
Quercitrin	1948.25 ± 105.21	104.94 ± 5.56	130.69 ± 7.32	655.64 ± 34.09	5134.61 ± 189.98
Luteolin	49117.9 ± 2652.37	1239.37 ± 65.69	23763.9 ± 1330.78	77047.5 ± 4006.47	40212.2 ± 1487.85
Naringenin	1268.73 ± 68.51	285.49 ± 15.13	1106.22 ± 61.95	15454 ± 803.61	2982.9 ± 110.37
Hesperetin	113.55 ± 6.13	610.96 ± 32.38	83.88 ± 4.70	1195.68 ± 62.18	nd

**Table 3:** Inhibitory activity of *α*-amylase and *α*-glucosidase of the methanolic extract of different parts of the plant *Vitex* (IC_50_).

Part of *Vitex*	*α*-Amylase inhibition	*α*-Glucosidase inhibition
Leaves	2156.80	3193.17
Roots	2357.30	3555.36
Stems	3152.11	3987.10
Flowers	2931.48	2921.84
Seeds	3260.60	2992.75
Acarbose	396.42	199.53

**Table 4:** Minimum Inhibitory Concentration (MIC) and bactericidal (CMB) concentration of the methanolic extract of the different parts of the *Vitex agnus-castus* plant.

Bacteria	Leaves			Roots			Stems			Flowers			Seeds			Ch		
	MIC	MBC	r	MIC	MBC	r	MIC	MBC	r	MIC	MBC	r	MIC	MBC	r	MIC	MBC	r
*B. subtilis*	15.62	15.62	1	31.25	31.25	1	15.62	15.62	1	15.625	31.25	2	15.62	15.62	1	0.05	0.05	1
*E. coli*	15.62	15.62	1	15.62	15.625	1	15.62	31.25	2	31.25	31.25	1	15.62	15.62	1	0.095	0.095	1
*P. aeruginosa*	15.62	15.62	1	15.62	15.625	1	15.62	15.62	1	7.81	7.81	1	15.62	15.62	1	0.095	0.095	1
*S. enterica*	7.81	15.62	2	31.25	31.25	1	7.81	15.62	2	7.81	15.62	2	7.81	15.62	2	0.05	0.05	1
*S. aureus*	15.62	15.62	1	31.25	31.25	1	15.62	15 .62	1	7.81	15.62	2	15.62	15.62	1	0.05	0.05	1

Ch: chloramphenicol.

## Data Availability

All used data in this study were cited in the manuscript.

## References

[1] Daniele C., Thompson Coon J., Pittler M. H., Ernst E. (2005). Vitex agnus castus. *Drug Safety*.

[2] Zahid H., Rizwani G. H., Ishaqe S. (2016). Phytopharmacological review on *Vitex agnus-castus*: a potential medicinal plant. *Chin. Herb. Med.*.

[3] Cerqueira R. O., Frey B. N., Leclerc E., Brietzke E. (2017). Vitex agnus castus for premenstrual syndrome and premenstrual dysphoric disorder: a systematic review. *Archives of Women's Mental Health*.

[4] Hoberg E., Meier B., Sticher O. (2001). Quantitative high performance liquid chromatographic analysis of casticin in the fruits of Vitex agnus-castus. *Pharmaceutical Biology*.

[5] Li S.-H., Zhang H.-J., Qiu S.-X. (2002). Vitexlactam A, a novel labdane diterpene lactam from the fruits of *Vitex agnus-castus*. *Tetrahedron Letters*.

[6] Sahib A. H. A., Al-Shareefi E., Hameed I. H. (2019). Detection of bioactive compounds of Vitex agnus-castus and Citrus sinensis using Fourier-transform infrared spectroscopic profile and evaluation of its anti-microbial activity. *Indian Journal of Public Health Research & Development*.

[7] Ricarte L. P., Bezerra G. P., Romero N. R. (2020). Chemical composition and biological activities of the essential oils from Vitex-agnus castus, Ocimum campechianum and Ocimum carnosum. *Anais da Academia Brasileira de Ciências*.

[8] Berrani A., Lrhorfi L. A., Bengueddour R. (2020). The effect of the methanol extracts of various parts of Vitex Agnus Castus on the corrosion of E24 steel in a neutral medium (NaCl 3.5 %). *Journal of Chemical Technology & Metallurgy*.

[9] Berrani A., Lrhorfi L. A., Larbi O. M. (2018). Hypoglycemic effect of Vitex agnus castus extract in diabetic rats induced by streptozotocin. *Phytothérapie*.

[10] Bakr R. O., Zaghloul S. S., Hassan R. A., Sonousi A., Wasfi R., Fayed M. A. A. (2020). Antimicrobial activity of Vitex agnus-castus essential oil and molecular docking study of its major constituents. *Journal of Essential Oil-Bearing Plants*.

[11] Enayat Gholampour T., Fadaei Raieni R., Pouladi M., Larijani M., Pagano M., Faggio C. (2020). The dietary effect of Vitex agnus-castus hydroalcoholic extract on growth performance, blood biochemical parameters, carcass quality, sex ratio and gonad histology in zebrafish (Danio rerio). *Applied Sciences*.

[12] Singleton V. L., Rossi J. A. (1965). Colorimetry of total phenolics with phosphomolybdic-phosphotungstic acid reagents. *American Journal of Enology and Viticulture*.

[13] Marmouzi I., Karym E. M., Saidi N. (2017). In vitro and in vivo antioxidant and anti-hyperglycemic activities of Moroccan oat cultivars. *Antioxidants*.

[14] Huang B., Ke H., He J., Ban X., Zeng H., Wang Y. (2011). Extracts of *Halenia elliptica* exhibit antioxidant properties *in vitro* and *in vivo*. *Food and Chemical Toxicology*.

[15] Riov J., Gottlieb H. E. (1980). Metabolism of auxin in pine tissues: indole-3-acetic acid conjugation. *Physiologia Plantarum*.

[16] Juma B. F., Majinda R. R. T. (2004). Erythrinaline alkaloids from the flowers and pods of *Erythrina lysistemon* and their DPPH radical scavenging properties. *Phytochemistry*.

[17] Sarker S. D., Nahar L., Kumarasamy Y. (2007). Microtitre plate-based antibacterial assay incorporating resazurin as an indicator of cell growth, and its application in the *in vitro* antibacterial screening of phytochemicals. *Methods*.

[18] Ponce A. G., Fritz R., del Valle C., Roura S. I. (2003). Antimicrobial activity of essential oils on the native microflora of organic Swiss chard. *LWT - Food Science and Technology*.

[19] Hirobe C., Qiao Z.-S., Takeya K., Itokawa H. (1997). Cytotoxic flavonoids from *Vitex agnus-castus*. *Phytochemistry*.

[20] Latoui M., Aliakbarian B., Casazza A. A., Seffen M., Converti A., Perego P. (2012). Extraction of phenolic compounds from *Vitex agnus-castus* L.. *Food and Bioproducts Processing*.

[21] Sogame M., Naraki Y., Sasaki T. (2019). Quality assessment of medicinal product and dietary supplements containing *Vitex agnus-castus* by HPLC fingerprint and quantitative analyses. *Chemical and Pharmaceutical Bulletin*.

[22] Ahangarpour A., Oroojan A. A., Khorsandi L., Najimi S. A. (2017). Pancreatic protective and hypoglycemic effects of *Vitex agnus-castus* L. fruit hydroalcoholic extract in D-galactose-induced aging mouse model. *Res. Pharm. Sci.*.

[23] Marmouzi I., Kharbach M., El Jemli M. (2019). Antidiabetic, dermatoprotective, antioxidant and chemical functionalities in *Zizyphus lotus* leaves and fruits. *Industrial Crops and Products*.

[24] Gill B. S., Mehra R., Navgeet S. K., Kumar S. (2018). Vitex negundo and its medicinal value. *Molecular Biology Reports*.

[25] Matsui T., Kobayashi M., Hayashida S., Matsumoto K. (2002). Luteolin, a flavone, does not suppress postprandial glucose absorption through an inhibition of *α*-glucosidase action. *Bioscience, Biotechnology, and Biochemistry*.

[26] Yan J., Zhang G., Pan J., Wang Y. (2014). *α*-Glucosidase inhibition by luteolin: kinetics, interaction and molecular docking. *International Journal of Biological Macromolecules*.

[27] Kim J.-S., Kwon C.-S., Son K. H. (2000). Inhibition of alpha-glucosidase and amylase by luteolin, a flavonoid. *Bioscience, Biotechnology, and Biochemistry*.

[28] Nicasio P., Aguilar-Santamaría L., Aranda E., Ortiz S., González M. (2005). Hypoglycemic effect and chlorogenic acid content in twoCecropia species. *Phytotherapy Research*.

[29] Park J.-S., Yang J.-S., Hwang B.-Y., Yoo B.-K., Han K. (2009). Hypoglycemic effect of Yacon tuber extract and its constituent, chlorogenic acid, in streptozotocin-induced diabetic rats. *Biomolecules & Therapeutics*.

[30] Oboh G., Agunloye O. M., Adefegha S. A., Akinyemi A. J., Ademiluyi A. O. (2015). Caffeic and chlorogenic acids inhibit key enzymes linked to type 2 diabetes (in vitro): a comparative study. *Journal of Basic and Clinical Physiology and Pharmacology*.

[31] Karthikesan K., Pari L., Menon V. P. (2010). Combined treatment of tetrahydrocurcumin and chlorogenic acid exerts potential antihyperglycemic effect on streptozotocin-nicotinamide-induced diabetic rats. *General Physiology and Biophysics*.

[32] Ong K. W., Hsu A., Tan B. K. H. (2013). Anti-diabetic and anti-lipidemic effects of chlorogenic acid are mediated by AMPK activation. *Biochemical Pharmacology*.

[33] Mittler R. (2002). Oxidative stress, antioxidants and stress tolerance. *Trends in Plant Science*.

[34] Uttara B., Singh A. V., Zamboni P., Mahajan R. T. (2009). Oxidative stress and neurodegenerative diseases: a review of upstream and downstream antioxidant therapeutic options. *Current Neuropharmacology*.

[35] Brand-Williams W., Cuvelier M. E., Berset C. (1995). Use of a free radical method to evaluate antioxidant activity. *LWT - Food Science and Technology*.

[36] Hajdú Z., Hohmann J., Forgo P. (2007). Diterpenoids and flavonoids from the fruits ofVitex agnus-castus and antioxidant activity of the fruit extracts and their constituents. *Phytotherapy Research*.

[37] Sağlam H., Pabuçcuoğlu A., Kıvçak B. (2007). Antioxidant activity ofVitex agnus-castusL. extracts. *Phytotherapy Research*.

[38] Gonçalves R., Ayres V. F. S., Carvalho C. E. (2017). Chemical composition and antibacterial activity of the essential oil of Vitex agnus-castus L. (Lamiaceae). *Anais da Academia Brasileira de Ciências*.

[39] Stojković D., Soković M., Glamočlija J. (2011). Chemical composition and antimicrobial activity of _Vitex agnus-castus_ L. fruits and leaves essential oils. *Food Chemistry*.

[40] Ghannadi A., Bagherinejad M., Abedi D., Jalali M., Absalan B., Sadeghi N. (2012). Antibacterial activity and composition of essential oils from Pelargonium graveolens L'Her and Vitex agnus-castus L. *Iranian Journal of Microbiology*.

[41] Bouyahya A., Dakka N., Et-Touys A., Abrini J., Bakri Y. (2017). Medicinal plant products targeting quorum sensing for combating bacterial infections. *Asian Pacific Journal of Tropical Medicine*.

[42] Naveed M., Hejazi V., Abbas M. (2018). Chlorogenic acid (CGA): a pharmacological review and call for further research. *Biomedicine & Pharmacotherapy*.

